# Are heat warning systems effective?

**DOI:** 10.1186/1476-069X-12-27

**Published:** 2013-04-05

**Authors:** Ghasem (Sam) Toloo, Gerard FitzGerald, Peter Aitken, Kenneth Verrall, Shilu Tong

**Affiliations:** 1 School of Public Health and Social Work, Queensland University of Technology, Victoria Park Road, Kelvin Grove, Brisbane, QLD 4059, Australia; 2 School of Public Health, Tropical Medicine and Rehabilitation Sciences, James Cook University, Townsville, QLD 4811, Australia; 3 Environmental and Resource Sciences Division, Department of Environment and Resource Management, Queensland Government, Brisbane, QLD 4001, Australia

**Keywords:** Heat warning system, Effectiveness, Mortality, Morbidity, Health beliefs, Health service utilization

## Abstract

Heatwaves are associated with significant health risks particularly among vulnerable groups. To minimize these risks, heat warning systems have been implemented. The question therefore is how effective these systems are in saving lives and reducing heat-related harm. We systematically searched and reviewed 15 studies which examined this. Six studies asserted that fewer people died of excessive heat after the implementation of heat warning systems. Demand for ambulance decreased following the implementation of these systems. One study also estimated the costs of running heat warning systems at US$210,000 compared to the US$468 million benefits of saving 117 lives. The remaining eight studies investigated people’s response to heat warning systems and taking appropriate actions against heat harms. Perceived threat of heat dangers emerged as the main factor related to heeding the warnings and taking proper actions. However, barriers, such as costs of running air-conditioners, were of significant concern, particularly to the poor. The weight of the evidence suggests that heat warning systems are effective in reducing mortality and, potentially, morbidity. However, their effectiveness may be mediated by cognitive, emotive and socio-demographic characteristics. More research is urgently required into the cost-effectiveness of heat warning systems’ measures and improving the utilization of the services.

## Background

Heatwaves kill thousands of people around the world each year [1-4]. In addition, more adverse health conditions including heat-related illnesses (e.g., heat stroke, dehydration, acute myocardial infarction) and heat exacerbated illnesses (e.g., renal disease, ischaemic heart disease and mental health disorders) are associated with heatwaves [5-9]. These effects are felt more severely among the vulnerable groups such as the elderly, children, people with pre-existing chronic conditions, as well as Culturally and Linguistically Diverse (CALD) communities, those living in poverty and isolation, homeless, and people with disabilities as they have reduced capacity and/or awareness to respond to temperature increases [6,10,11].

With higher frequency of heatwaves projected to occur as a result of climate change [12], locally tailored action plans are implemented in many affected areas to minimize harm to those most at risk. These plans which may include early alerts and advisories and a variety of emergency measures to mitigate the heat dangers, are called “heat warning systems” (HWS) or “heat health warning systems” (HHWS) [13,14]. Given the variations in the type and extent of measures implemented as part of an HWS, it is difficult to evaluate the effectiveness of these systems in terms of saving lives and reducing heat-related harms. However, it is important to do so in order to improve decision making in public health responses, to determine cost-benefits, and to determine which mitigation and adaptation strategies are most effective in reducing heat-related morbidity and mortality. Similarly the systems need to consider whether target populations are reached and whether improvements are observed as a result of implementing HWS in knowledge, awareness, service utilization, and heat-health behavior changes.

As part of our study on the evaluation of the effectiveness of heat warning systems, we conducted a structured search in major databases and systematically retrieved and reviewed 15 articles identified using pre-defined selection criteria. This commentary provides an overview of the findings, research limitations and challenges, and formulates recommendations for further research directions.

### Effectiveness of HWS

#### HWS and reduction in adverse health conditions

Six articles asserted that among the populations studied, the number of expected deaths reduced after the HWS implementation compared to before that [15-20]. One study was inconclusive [21]. Due to different population and sub-group characteristics (such as age or disease groups) we were unable to estimate the extent of effectiveness. For instance, one study estimated that nearly 4400 excess deaths were avoided in France as a result of implementing HWS in 2006 [20], while another calculated a reduction of approximately 1300 ischemic and stroke related deaths among the people aged 65+ following the HWS implementation in Hong Kong [17]. To date, no studies have directly examined the impact of HWS on morbidity. However, one study reported that in Milwaukee, Wisconsin, the dispatch of emergency medical services (as a proxy indicator for morbidity) was reduced by 49%-73% on heatwave days with an alert system in 1999 compared to 1995 without HWS [17]. In terms of cost-benefits of HWS, only one study estimated that the US$210,000 cost of running HWS was highly cost-effective compared to the US$468 million benefits of saving 117 lives [18].

#### HWS and human response

Protective measures in response to heatwaves can range from temporary programs such as warnings and advisories, cooling shelters and “buddy” checks, to long term plans such as improving buildings and living environments [22-24]. However, the effectiveness of these, particularly the short term, measures depend on how well they are heeded and adopted by the target groups. We found eight papers addressing these issues. The consistent theme from these studies was that people who perceived themselves personally vulnerable were more likely to protect themselves such as using air conditioners, hydrating, dressing lightly, and avoiding strenuous activities [25-31]. However, a qualitative study of people aged 75+ found that most participants did not consider themselves vulnerable to or threatened by heat [30] while, interestingly, these participants considered other people of the same age group as vulnerable but not themselves.

## Discussion

The evidence reviewed in this study as well as descriptive and speculative studies [32,33] suggest that implementing HWS is associated with lower mortality, but more research is needed to assess the impact of implementing HWS on morbidity. Only one study did a cost-benefit analysis and found that the benefits of saving lives far outweighed the costs of running HWS. It is nonetheless important to evaluate the effectiveness of the intervention strategies in order to maximize their benefits to the target vulnerable groups, but none of the previous studies have examined this issue. Consistent with the previous work by Bassil and Cole [34], our findings further advance the knowledge and debate in this area by drawing attention to the limitations and challenges facing studies of the HWS effectiveness.

### Methodological challenges

In order to estimate the effectiveness of implementing HWS, all the studies need to compare the impacts of at least two similar heatwaves: one with a HWS and another without a HWS. However, these estimates are subject to major methodological and analytical challenges. To accurately estimate the effectiveness of a HWS, an accurate estimate of the net impact of heat on mortality or morbidity is required for both heatwave periods. Such estimates can be complicated as many other factors exacerbate or alleviate the impact of a heat event, such as timing, severity, duration of the heatwave, and night-time temperature, as well as socio-demographic characteristics, population acclimatization, prevalence of chronic disease, urban heat island effect, indoor air quality and potentially ozone and humidity. Additionally events that can occur alongside heatwaves such as power blackouts, transportation failures, drought and fires can also impact on morbidity and mortality [6,12,24,35-37].

Inclusion of all these factors in one analytical model may be impossible due to availability of data and complexity of the analytical techniques. This makes assessing the effectiveness of the adaptation and mitigation strategies in the HWS problematic. The identified studies used different techniques to overcome these problems. Some examined the difference between “expected” and “observed” excess mortality associated with one heatwave compared to the most recent previous heatwave event [18,20]; two compared the difference between the average number of deaths and ambulance use observed during two heatwave periods [16,19]; and others used a combination of statistical techniques to estimate the numbers or odds of excess mortality during heatwaves with or without HWS. Only four studies adjusted their analytical models for some of the above-mentioned confounding factors [16-19]. Given the relatively crude techniques used to estimate the excess impacts, the results from the assessment of the effectiveness of HWS relying on these methods should be interpreted cautiously.

In addition to the factors mentioned above, other factors may intervene or confound the findings such as improvements in health care and living conditions including the use of air-conditioners and heat insulating building materials. However, an absence of reduction in mortality in future heatwaves is unlikely to simply indicate ineffectiveness of HWS without careful consideration of the impact of changing population characteristics. For instance, an increase in the number of vulnerable populations (e.g. the elderly, and those with pre-existing medical conditions), or population growth conducive to building density and risk of urban heat island effect, may exacerbate the impact of heat events.

Different studies are often not directly comparable due to different designs, locations and population characteristics. Because of the complexity of the HWS interventions, it is difficult to determine which components are contributing to the HWS effectiveness directly and/or indirectly. Additionally, people naturally change their behavior in summertime, which may give them a sense of preparedness and immunity, hence not feeling the need to take a particular action to heat warnings [26,27]. Other relevant health campaigns, such as the long-running “Slip Slop Slap Seek Slide” sun protection campaign in Australia [38], which provides advice on ways of reducing heat exposure during the hotter hours of the day, may also have co-beneficial effects on preparing people to protect themselves against heat stress. Nevertheless, heatwaves affect the vulnerable groups rapidly and severely. Therefore, relying on “common sense” and personal feeling of heat may prevent them from seeking timely assistance.

### Benefitting from theoretical frameworks

Use of theoretical frameworks such as the Health Belief Model, Health Service Utilization Model [39] or Precaution Adoption Process [40] can enhance our understanding and interpretation of human behavior. On the other hand, lack of a conceptual framework, particularly in quantitative surveys, can lead to the collection of a number of “interesting” information, but miss “crucial” information, further undermining the research. For instance, despite the importance of using air-conditioners in reducing heat impacts, only two studies reported on the cost of running air-conditioning as a concern to a considerable number of the participants [27,29], and even then it was not clear if the concern was actually associated with using the air-conditioners during heatwaves. In our review, only one study mentioned the application of the Health Belief Model [29] in guiding their research, and another study [41] applied a framework of Awareness-Knowledge-Practice that seems to be a modified version of the Knowledge-Attitude-Practice (KAP) model recommended by World Health Organization. Other studies used variables and indicators that resonated with some elements of the mentioned theories, but none clearly based their research on any cohesive conceptual framework.

## Conclusions

The existing evidence supports the notion that HWS are effective in reducing heat-related mortality (and potentially morbidity). However, the small number of the studies to date, as well as methodological and theoretical concerns call for further and more robust research to provide a strong evidence-base to allow the evaluation of the effectiveness of HWS in terms of reaching the target populations, changing behaviors and reducing adverse heat impacts. Future studies are required to evaluate the effectiveness of HWS with regards to heat-related and heat-exacerbated morbidity, particularly on conditions such as respiratory, cardiovascular, renal and diabetic conditions [5,42]. More advanced analytical techniques are needed to control the effects of confounding variables and to provide more accurate estimates. Further research is also needed to establish which measures and programs are more cost-effective in reducing the adverse health impacts. Additionally, more research is urgently required into mechanisms of improving the utilization of services by the vulnerable populations and groups during heatwaves in both the developed and developing societies.

## Abbreviations

CALD: Culturally and Linguistically Diverse; HWS: Heat warning systems; HHWS: Heat health warning systems; KAP: Knowledge-Attitude-Practice

## Competing interests

At the time of conducting the study, KV was a Principal Scientist at the Queensland Department of Environment and Resources Management, which partly funded this project. The department has since been restructured and KV is now affiliated with the Queensland Department of Science, Information Technology, Innovation and Arts**.**

## Authors’ contributions

GT and ST contributed to the conception, design, data search and analysis, interpretation, drafting and revising the paper. GF, PA and KV contributed to the conception, analysis and interpretation, and revision of the paper. All authors critically reviewed and approved the manuscript for publication.
